# Altered Structural Correlates of Impulsivity in Adolescents with Internet Gaming Disorder

**DOI:** 10.3389/fnhum.2016.00004

**Published:** 2016-01-28

**Authors:** Xin Du, Xin Qi, Yongxin Yang, Guijin Du, Peihong Gao, Yang Zhang, Wen Qin, Xiaodong Li, Quan Zhang

**Affiliations:** ^1^Department of Radiology and Tianjin Key Laboratory of Functional Imaging, Tianjin Medical University General Hospital Tianjin, China; ^2^Department of Psychology, Linyi Fourth People’s Hospital, Linyi Shandong Province, China; ^3^Department of Radiology, Linyi People’s Hospital, Linyi Shandong Province, China

**Keywords:** internet gaming disorder, impulsivity, gray matter volume, voxel-based morphometry, adolescent

## Abstract

Recent studies suggested that internet gaming disorder (IGD) was associated with impulsivity and structural abnormalities in brain gray matter (GM). However, no morphometric study has examined the association between GM and impulsivity in IGD individuals. In this study, 25 adolescents with IGD and 27 healthy controls (HCs) were recruited, and the relationship between Barratt impulsiveness scale-11 (BIS) score and gray matter volume (GMV) was investigated with the voxel-based morphometric (VBM) correlation analysis. Then, the intergroup differences in correlation between BIS score and GMV were tested across all GM voxels. Our results showed that the correlations between BIS score and GMV of the right dorsomedial prefrontal cortex (dmPFC), the bilateral insula and the orbitofrontal cortex (OFC), the right amygdala and the left fusiform gyrus decreased in the IGD group compared to the HCs. Region-of-interest (ROI) analysis revealed that GMV in all these clusters showed significant positive correlations with BIS score in the HCs, while no significant correlation was found in the IGD group. Our findings demonstrated that dysfunction of these brain areas involved in the behavior inhibition, attention and emotion regulation might contribute to impulse control problems in IGD adolescents.

## Introduction

Internet addiction is a rapidly growing concern in the world and is associated with a variety of psychiatric disorders (Ko et al., 2012). Young (1998) defined Internet addiction, including internet gaming disorder (IGD), as an impulse control disorder. Prior studies observed that subjects with internet addiction showed higher impulsivity compared to healthy controls (HCs; Cao et al., 2007; Lee et al., 2012). In addition, impulsivity was also noted to predict internet use disorder in the longitudinal studies (Billieux et al., 2011; Gentile et al., 2011). Furthermore, adolescents with IGD often exhibit behavioral control difficulties during performing the executive or impulse control related tasks (Cao et al., 2007; Ko et al., 2009; Dong et al., 2010, 2011; Dong et al., 2013a,b; Zhou et al., 2012; Dong and Potenza, 2014). Given that impulsive behavior may lead to serious impairments in psychological and social functions, such as suicide attempts and crime, it is necessary to investigate the neural substrates of the higher impulsivity in IGD adolescents.

Functional neuroimaging studies (Dong et al., 2012, 2013a,b, 2014; Liu et al., 2015) demonstrated that the subjects with IGD had aberrant activations in frontal, insular, temporal and parietal cortex compared with the HCs during performing the impulse control related tasks, and the activations in anterior cingulated cortex and insula significantly correlated to the correct incongruent trial reaction time and subjective experience to lose (Dong et al., 2012, 2013a). Previous structural studies have revealed that IGD was associated with structural abnormalities in gray matter (GM), such as decreased gray matter volume (GMV) in frontal, cingulated, insular, parietal cortex and amygdala, and increased GMV in temporal and parahippocampal cortex (Yuan et al., 2011; Hong et al., 2013; Kühn and Gallinat, 2014, 2015; Kühn et al., 2014; Sun et al., 2014; Ko et al., 2015). Recently, accumulating neuroimaging studies investigated structural correlates of impulsivity and revealed heterogeneous findings in healthy subjects and other impulsivity-related disorders. In healthy subjects, negative (Boes et al., 2009; Matsuo et al., 2009; Schilling et al., 2012, 2013) or positive (Gardini et al., 2009; Schilling et al., 2012; Cho et al., 2013) correlations were both reported between impulsivity and GMV/cortical thickness in frontal, temporal regions and amygdala. The significant correlations between GMV in the orbitofrontal cortex (OFC)/amygdala and impulsivity were also found in patients with major depressive disorder, alcoholism, attention-deficit/hyperactivity disorder, posttraumatic stress disorder, antisocial personality disorder and bipolar disorder (Antonucci et al., 2006; Tajima-Pozo et al., 2015). However, the relationship between impulsivity and GMV in IGD adolescents was largely unknown.

In this study, we aimed to identify altered structural correlates of impulsivity using a voxel-based morphometry (VBM) analysis in IGD adolescents compared to the HCs. Twenty-five male IGD adolescents and 27 age-, and education-matched HCs were recruited and impulsivity was evaluated with the Barratt impulsiveness scale-11 (BIS). Exploring the relationship between impulsivity and GMV in IGD adolescents may provide new insights into the underlying neural mechanisms of the higher impulsivity in IGD adolescents.

## Materials and Methods

### Subjects

Twenty-five right-handed male adolescents with IGD were recruited in this study. Only the male subjects were examined because of the relatively small number of females with internet gaming experience. The inclusion criteria for IGD group were: (i) subjects with five or more “yes” responses on the Young Diagnostic Questionnaire for Internet addition (Young, 1998); (ii) online game playing time ≥4 h per day; and (iii) Young’s 20-item internet addiction test (IAT) score ≥50. Twenty-seven right-handed, age-, and education-matched male healthy adolescents were recruited as the HCs. The inclusion criteria for the HCs included: (i) the subjects had not reached the diagnostic criteria of Young Diagnostic Questionnaire for Internet addition; (ii) online game playing time ≤2 h per day; and (iii) Young’s 20-item IAT score <50. The exclusion criteria for both groups were: (i) existence of a neurological disorder; (ii) alcohol or drug abuse; and (iii) any physical illness such as a brain tumor, brain trauma or epilepsy as assessed according to clinical evaluations and medical records. Intelligence Quotient (IQ) of all participants was tested using standard Rawen’s progressive matrices. The detailed demographic information was shown in Table 1. The protocol of this study was approved by the Ethical Committee of Tianjin Medical University General Hospital, and written informed consent was obtained from all participants or their guardians according to institutional guidelines.

**Table 1:** **Participant’s characteristics for IGD group and the HCs**.

	IGD	HCs
Item	*N* = 25	*N* = 27	*T*	*P*
Age (years)	17.28 ± 3.42	17.48 ± 2.87	−0.231	0.819
Education (years)	10.40 ± 2.71	11.33 ± 2.95	−1.186	0.241
IQ	50.08 ± 7.27	47.89 ± 6.17	1.175	0.246
Online game playing time (hours/day)	8.56 ± 4.67	1.43 ± 0.63	7.860	<0.001
IAT Score	69.96 ± 11.43	32.15 ± 7.56	14.172	<0.001
BIS Score	68.56 ± 11.42	55.33 ± 7.87	4.895	<0.001

*BIS, Barratt impulsiveness scale-11; IAT, internet addiction test; IQ, Intelligence Quotient*.

### Impulsivity Assessment

The BIS, a self-report questionnaire designed to measure impulsivity (Patton et al., 1995), was used to measure impulsivity of all participants. All items were answered on a 4-point Likert-scale (Rarely/Never; Occasionally; Often; Almost always/Always). Higher score signifies higher impulsivity.

### Structural MRI

MR imaging was conducted on a Siemens 3.0T scanner (Magnetom Verio, Siemens, Erlangen, Germany). A T1-weighted volumetric magnetization-prepared rapid gradient-echo sequence was used to acquire a series of 192 contiguous sagittal high resolution anatomical images with the following parameters: TR = 2000 ms, TE = 2.34 ms, TI = 900 ms, flip angle = 9°, FOV = 256 mm × 256 mm, slice thickness = 1 mm, matrix size = 256 × 256.

### Voxel-Based Morphometry (VBM) Analysis

Structural images were preprocessed using VBM8 tool-box^1^ of the SPM8 (Wellcome Department of Imaging Neuroscience, London, UK; available at http://www.fil.ion.ucl.ac.uk/spm/software/spm8 implemented on MATLAB R2010a (Math Works Inc., Sherborn, MA, USA). Three-dimensional geometric correction was performed during reconstruction of the images. Then, the individual native images of all participants were segmented into GM, white matter, and cerebral spinal fluid, and the GM segments were normalized to the Montreal Neurological Institute template. Next, the normalized GM segments were registered to a template generated from their own mean by diffeomorphic anatomical registration through exponentiated lie algebra (DARTEL). The registered partial volume images were then modulated by dividing the Jacobian of the warp field to correct for local expansion or contraction. Final modulated GM images were smoothed with an isotropic Gaussian kernel of 8 mm full-width at half maximum. To exclude from the statistical analysis pixels assigned by the segmentation to GM with low probability values and pixels with a low inter-subject anatomical overlay after normalization, the mean image of normalized GM from all subjects was used to create a GM mask, whose threshold was set at a value of 0.30 (pixels with computed GM fraction values >30% were selected) and then used as an explicit mask for the statistical analysis.

### Statistical Analysis

The intergroup differences in age, education, IQ, online game playing time (hours/day), IAT score and BIS score were compared using a two-sample *t*-test in SPSS 18.0 and the significance level was set at *p* < 0.05.

To characterize if the correlations between GMV and BIS score are different between the two groups, we introduced a general linear model considering GMV as dependent variable, with group (the HCs vs. IGD), BIS score and their interaction as interested independent variables and the age as confounding variable (Giedd and Rapoport, 2010). The BIS score of each subject was demeaned in each group before entering into the GLM model. The parameter (also called the regression coefficient) between the GMV and BIS score of each group of each voxel was estimated, and the regression coefficients between the HCs and IGD group were compared using *t*-test. Given that our study is an exploratory research and entails a small sample size, a relatively loose significance threshold (uncorrected *p* < 0.005; cluster size >200 voxels) was used here.

Clusters with altered correlations between GMV and BIS score in IGD adolescents were defined as regions of interest (ROIs). Average GMV in the ROIs were extracted and the correlations between average GMV of these ROIs and BIS score were further tested using the Pearson correlation analysis in SPSS 18.0. ROI-wise intergroup comparisons of average GMV of these ROIs were also performed using two-sample *t*-test. The significance level was set at *p* < 0.05.

## Results

### Demographic Data Results

There was no significant intergroup difference in age, education and IQ. Online game playing time (hours/day), IAT score and BIS score were significantly higher in the IGD group than in the HCs (Table 1).

### Voxel-Wise Correlation Results

**Figure 1:**
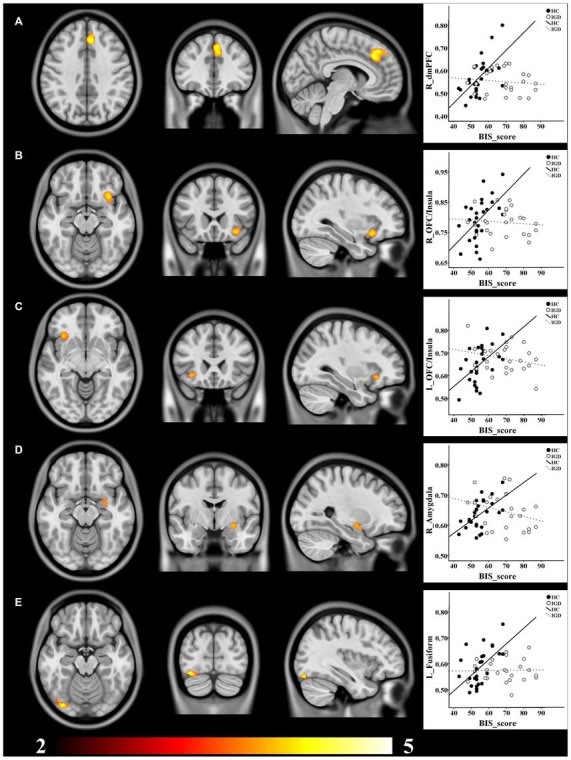
**Brain regions showing decreased structural correlates of impulsivity in IGD adolescents compared to the HCs. (A)** dmPFC; **(B)** right OFC/Insula; **(C)** left OFC/Insula; **(D)** right Amygdala; **(E)** left Fusiform. GMV of all these clusters showed positive correlations with BIS score in the HCs, while no significant correlation was found in the IGD group. *T* value ranges from 2 to 5 presented by color bar.

The voxel-wise correlation analysis revealed that, compared to the HCs, IGD adolescents had lower correlations between BIS score and GMV in the right dorsomedial prefrontal cortex (dmPFC), the bilateral OFC/insula, the right amygdala and the left fusiform cortex (uncorrected *p* < 0.005; cluster size >200 voxels; Table 2, Figure 1).

**Table 2:** **Regions showing decreased structural correlates of impulsivity in adolescents with IGD compared with the HCs**.

	Peak MNI Coordinates				Cluster Size (voxels)
Region	*X*	*Y*	*Z*	*T* value*
R_dmPFC	6	32	36	4.04	926
R_OFC/insula	33	18	−14	3.68	425
L_OFC/insula	−33	27	−9	3.37	213
R_Amygdala	30	−6	−12	3.36	227
L_Fusiform	−35	−84	−17	4.08	342

*Uncorrected P < 0.005, Cluster size >200. dmPFC, dorsomedial prefrontal cortex; L, left; OFC, orbitofrontal cortex; R, right.

### Region-Of-Interest (ROI) Correlation Results

ROI-based correlation analysis showed significant positive correlations between GMV of all these clusters and BIS score in the HCs, while no significant correlation was found in the IGD group (Table 3, Figure 1).

**Table 3:** **The correlations between the GMV of ROIs and BIS score in IGD adolescents and the HCs**.

ROI	IGD		HCs	
	*r*	*P*	*r*	*P*
R_dmPFC	−0.121	0.565	0.608	0.001
R_OFC/insula	−0.103	0.624	0.541	0.004
L_OFC/insula	−0.255	0.218	0.517	0.006
R_Amygdala	−0.288	0.162	0.564	0.002
L_Fusiform	0.250	0.905	0.554	0.003

*dmPFC, dorsomedial prefrontal cortex; L, left; OFC, orbitofrontal cortex; R, right*.

### Region-Of-Interest (ROI) Gray Mater Volume (GMV) Results

There was no significant intergroup difference in GMV within the right dmPFC, the bilateral OFC/insula, the right amygdala and the left fusiform cortex (Table 4).

**Table 4:** **Comparison of GMV within the ROIs between IGD adolescents and the HCs**.

ROI	IGD	HCs	*T*	*P*
R_dmPFC	0.552 ± 0.053	0.571 ± 0.081	−0.981	0.331
R_OFC/insula	0.783 ± 0.044	0.793 ± 0.070	−0.622	0.537
L_OFC/insula	0.676 ± 0.061	0.650 ± 0.081	1.331	0.189
R_Amygdala	0.647 ± 0.059	0.636 ± 0.047	0.732	0.467
L_Fusiform	0.575 ± 0.042	0.585 ± 0.070	−0.647	0.521

*dmPFC, dorsomedial prefrontal cortex; L, left; OFC, orbitofrontal cortex; R, right*.

## Discussion

In the present study, the correlation between GMV and impulsivity was investigated in adolescents with IGD. Altered correlations between impulsivity and GMV in the right dmPFC, the bilateral insula/OFC, the right amygdala and the left fusiform gurus were revealed in IGD adolescents compared to the HCs.

A number of neuroimaging studies revealed that the OFC and the dmPFC not only played a critical role in behavior inhibition but were also involved in the regulation of emotion (Horn et al., 2003; Kringelbach and Rolls, 2004; Ochsner et al., 2004; Rolls, 2004; Amodio and Frith, 2006; Lemogne et al., 2012). Previous fMRI studies showed significant activation of the OFC during response inhibition in healthy subjects, which positively correlated to trait impulsivity score (Brown et al., 2006; Goya-Maldonado et al., 2010). Patients with alcohol dependence also showed altered functional activation in the OFC during a stop signal task, which was associated with less control of impulsivity and emotion instability (Li et al., 2009). Neuroimaging study demonstrated that GMV of the dmPFC had a significant positive correlation with novelty seeking which refers to an individual’s tendency to action behaviors in healthy subjects (Gardini et al., 2009). It has also been reported that the dmPFC showed abnormal activation during performing cognitive task which contributed to self-regulation and impulse control processing in the subjects with IGD compared with healthy subjects (Meng et al., 2015). In addition, Cho et al. (2013) and Antonucci et al. (2006) reported that GMV of the dmPFC and the OFC positively correlated with BIS score in healthy subjects and a group of non-psychotic psychiatric clients, respectively. In line with these studies, our study also revealed positive correlations between BIS score and GMV of the right dmPFC and the bilateral OFC in the HCs. However, no significant correlation was found between impulsivity and GMV of the right dmPFC and bilateral OFC in IGD adolescents. These results implicated that the higher impulsivity in IGD adolescents was associated with the functional or the structural changes in the dmPFC and the OFC which are involved in behavior inhibition and emotion regulation.

In our study, the bilateral insula showed altered morphological correlations with impulsivity in IGD group. Insula belongs to the salience network (Di Martino et al., 2009; Menon and Uddin, 2010; Cauda et al., 2011; Deen et al., 2011; Menon, 2011) and is critical to the high-level cognitive control and attention processing (Menon and Uddin, 2010; Sharp et al., 2010). Horn et al. (2003) reported that trait impulsivity score was positively associated with activation of the insula in healthy subjects. Significant activations of the insula were also found in individuals with IGD during performing the cognitive tasks compared to healthy subjects (Dong et al., 2013a; Dong and Potenza, 2014). Furthermore, functional connectivity analysis revealed that the insula exhibited enhanced rest-stating functional connectivity with brain areas (including anterior cingulated cortex, putamen, angular gyrus, precuneous, precentral gyrus and supplemental motor area) which were involved in salience, self-monitoring, attention and movement control in IGD subjects (Zhang et al., 2015). These results indicated that abnormal salience network might contribute to the dysregulation of cognitive control and attention processing, which leaded to the higher impulsivity in IGD subjects.

In this study, altered structural correlations to impulsivity were found in the right amygdala and the left fusiform in the IGD adolescents. The amygdala was a critical region for regulating affective control and emotional/social behavior (Cisler and Olatunji, 2012; Gabard-Durnam et al., 2014). In addition, the amygdala was also a critical neural substrate for impulse control in patients with substance abuse (Hill et al., 2001). A recent study demonstrated that GM density of the bilateral amygdala decreased and connectivity between the prefrontal cortex/insula and the amygdala increased in IGD individuals, which might represent their emotion dysregulation (Ko et al., 2015). Additionally, the fusiform gyrus is mainly involved in processing of the emotion perception in facial stimuli and is also critical to emotion processing (Weiner et al., 2014). Taken together, it is plausible to postulate that altered emotion regulation may contribute to the higher impulsivity in IGD adolescents.

In our study, the positive correlations between impulsivity and GMV in the HCs may be related to stronger contribution of these brain areas to impulsive control. The individuals with higher impulsivity need to make more efforts to control their behaviors, and as a physiological compensatory response (Cho et al., 2013), GMV of the brain areas related to impulse control increased. Contrary to the HCs, no significant correlation was found in the IGD adolescents, which may be explained as the compensatory mechanism that invoked in the HCs was not presented in the IGD adolescents. However, it should be mentioned that there was no significant intergroup difference in GMV of the right dmPFC, the bilateral OFC/insula, the right amygdala and the left fusiform cortex, which may indicate that the IGD adolescents enrolled in our study were still at the early stage of IGD and the structural alterations were too subtle to be detected with VBM method. Moreover, it is difficult to determine whether disappeared correlations in the IGD adolescents was because of preexisting abnormal structural development or secondary to the IGD with this cross-sectional study. A longitudinal study may be helpful in clarifying this causality. Other limitations should also be noted in this study. First, as few women or other age groups exhibit IGD, only young males were recruited in our study. The current findings should be considered as specific to young males with IGD, and future studies should be performed in female subjects and in other age groups. Second, the relatively small sample size limited the statistical power; the results should be confirmed by a further study with a larger sample size.

In conclusion, the altered correlations between impulsivity and the GMV in the dmPFC, OFC, insula, amygdala and the fusiform in IGD adolescents indicated that the dysregulation in the brain networks involved in behavior inhibition, attention and emotion regulation might contribute to the high impulsivity in IGD adolescents.

## Author Contributions

XD, YY, XL and QZ designed research; XD, XQ, PG, YZ, GD and QZ performed research; YY, PG was involved in the clinical assessment; XD, YZ, GD, WQ, and QZ analyzed data; XD, YZ, XL, YY and QZ wrote the article.

## Conflict of Interest Statement

The authors declare that the research was conducted in the absence of any commercial or financial relationships that could be construed as a potential conflict of interest.

## Footnotes

^1^http://dbm.neuro.uni-jena.de/vbm/

## Abbreviations

BIS: Barratt impulsiveness scale-11
dmPFC: fractional anisotropy
GM: gray matter
GMV: gray matter volume
HCs: healthy controls
IAT: internet addiction test
IGD: internet gaming disorder
IQ: Intelligence Quotient
OFC: orbitofrontal cortex
ROI: region of interest
VBM: voxel-based morphometric.
