# Corrigendum: Altered Structural Correlates of Impulsivity in Adolescents with Internet Gaming Disorder

**DOI:** 10.3389/fnhum.2019.00124

**Published:** 2019-04-09

**Authors:** Xin Du, Xin Qi, Yongxin Yang, Guijin Du, Peihong Gao, Yang Zhang, Wen Qin, Xiaodong Li, Quan Zhang

**Affiliations:** ^1^Department of Radiology and Tianjin Key Laboratory of Functional Imaging, Tianjin Medical University General Hospital, Tianjin, China; ^2^Department of Psychology, Linyi Fourth People's Hospital, Linyi, Shandong Province, China; ^3^Department of Radiology, Linyi People's Hospital, Linyi, Shandong Province, China

**Keywords:** internet gaming disorder, impulsivity, gray matter volume, voxel-based morphometry, adolescent

In the original article, there was an error. The statement of the written informed consent procedures was incorrect because of inappropriate wording.

A correction has been made to the **Materials and Methods**, subsection **Subjects**, paragraph one:

“The protocol of this study was approved by the Ethical Committee of Tianjin Medical University General Hospital, and written informed consent was obtained from all participants or their guardians according to institutional guidelines.”

The authors apologize for this error and state that this does not change the scientific conclusions of the article in any way. The original article has been updated.

